# The Neural Circuitry of Reward Processing in Complex Social Comparison: Evidence from an Event-Related fMRI Study

**DOI:** 10.1371/journal.pone.0082534

**Published:** 2013-12-10

**Authors:** Xue Du, Meng Zhang, DongTao Wei, Wenfu Li, Qinglin Zhang, Jiang Qiu

**Affiliations:** 1 Faculty of Psychology, Southwest University, Chongqing, China; 2 School of Psychology, Southwest University, Chongqing, China; 3 Department of Psychology, Xinxiang Medical University, Xinxiang, Henan, China; Institute of Psychology, Chinese Academy of Sciences, China

## Abstract

In this study, Functional magnetic resonance imaging (fMRI) was conducted to investigate the mechanisms by which the brain activity in a complex social comparison context. One true subject and two pseudo-subjects were asked to complete a simple number estimate task at the same time which including upward and downward comparisons. Two categories of social comparison rewards (fair and unfair rewards distributions) were mainly presented by comparing the true subject with other two pseudo-subjects. Particularly, there were five conditions of unfair distribution when all the three subjects were correct but received different rewards. Behavioral data indicated that the ability to self-regulate was important in satisfaction judgment when the subject perceived an unfair reward distribution. fMRI data indicated that the interaction between the ventral striatum and the prefrontal cortex was important in self-regulation under specific conditions in complex social comparison, especially under condition of reward processing when there were two different reward values and the subject failed to exhibit upward comparison.

##  Introduction

Social comparison is considered as an important link between social context and self-evaluation [1]. Such comparison is also described as a central and ubiquitous phenomenon in human societies [2,3]. Studies have further shown that social comparison affects reward processing and is important for self-evaluation and maintenance, life satisfaction, and subjective well-being [2,4-6]. Other studies have also reported a close link between social comparison and individual depression, indicating the importance of social comparison as a component of stress treatment [1,7-9]. For instance, Vander et al. [10] indicated that individuals tend to compare themselves with others who may be superior in some ways to improve self-image. However, negative emotions (such as depression and low self-esteem) arise from upward comparison, which occurs when such individuals feel threatened by these superior people. Therefore, downward comparison is also common [7] because this response represents a stress-coping mechanism that negatively events affects the well-being of individuals [11,12].

Inevitable experiences, such as unfair events in life, are often associated with negative emotions in our life. Thereby, to adapt well in society, the brain processes these negative emotions, probably diverting from upward comparison to downward comparison. However, the mechanisms how this change occurs are yet to be elucidated. Consequently, a complex comparative paradigm including two confederates rather than one will be more real. 

Functional magnetic resonance imaging (fMRI) has shown that the ventral striatum and the ventromedial prefrontal cortex (PFC) are sensitive brain regions that can predict and register reward [13-16]. Tricomi et al. [13] also found that the ventral striatum and the ventromedial PFC functions in inequality-averse models of social preferences. 

The PFC located in the anterior part of the frontal lobes influences emotional behavior, social behavior, and decision making [17]. Moreover, PFC is a large area encompassing many sub-regions such as ventral and lateral PFC. Schultz [18] suggested that the PFC is involved in reward processing and classical conditioning. A single neuronal study has shown that the PFC in macaque becomes activated when reacts to different levels of expected rewards. For example, many neurons in PFC respond more frequently when macaques expect a larger reward [19]. Bault et al. [16] also found that the striatum and the medial PFC are more sensitive to social gains, and striatal activity associated with social gains predicts medial PFC activity during social choices. Haber and Knutson [20] pointed out that the superior frontal gyrus is important in reward processing when the working memory is required to monitor incentive-based behavioral responses. 

This study was based on a simple number estimation task similar to that used by Fliessbach et al. [21]. Fliessbach et al. [21] showed that the effect of relative comparisons is independent of the level of reward (high or low). In the present study, reward distribution was manipulated (Table 1) to investigate the mechanism of reward processing in the brain during a complex social comparison. This study hypothesized that the PFC modulates the reward circuitry when a subject receives an unfair reward in relation to other participants, although all of the participants performed the task equally well. This procedure likely results in a change in the direction of comparison especially during the conditions that there were two different reward values between the true and the other two pseudo-subjects. This change prevents further damage from negative emotions, and allows the subject to be relatively satisfied with reward distribution.

**Table 1:** Reward conditions.

Accuracy	Payoffs in CNY Subject-Pseudo A-Pseudo B	Condition
Subject correct	120 – 0 – 0	C1
	180 – 0 – 0	
	240 – 0 – 0	
Both Pseudo A and B correct	0 – 60 – 60	C2
	0 – 90 – 90	
	0 – 120 – 120	
Other	0 – 0 – 0	C3
	0 – 120 – 0 / 0 – 0 – 120	
	60 – 60 – 0 / 60 – 0 – 60	
All the three correct	40 – 40 – 40	C4
	60 – 60 – 60	
	80 – 80 – 80	
	20 – 50 – 50	C5
	30 – 75 – 75	
	40 – 100 – 100	
	20 – 40 – 60 / 20 – 60 – 40	C6
	30 – 60 – 90 / 30 – 90 – 60	
	40 – 80 – 120 / 40 – 120 – 80	
	80 – 20 – 20	C7
	120 – 30 – 30	
	160 – 40 – 40	
	60 – 40 – 20 / 60 – 20 – 40	C8
	90 – 60 – 30 / 90 – 30 – 60	
	120 – 80 – 40 / 120 – 40 – 80	
	40 – 20 – 60 / 40 – 60 – 20	C9
	60 – 30 – 90 / 60 – 90 – 30	
	80 – 40 – 120 / 80 – 120 – 40	

## Methods

### Ethics statement

This study has been approved by the IRB at Southwest China University. We had obtained appropriate ethics committee approval for the research reported, and all subjects gave written informed consent in our experiment. The study was approved by Southwest University Brain Imaging Center Institutional Review Board in accordance with the Declaration of Helsinki (1991). 

### Subjects

Nineteen right-handed, healthy males (mean age =21.2 years) participated in this study. Only male subjects were included to avoid confusion related to potential gender specific differences in social behavior and reward processing [21-23]. All of the subjects provided a written informed consent. The subjects did not suffer from past neurological or psychiatric illnesses, but have normal or corrected-to-normal vision. 

### Task and procedure

One true subject and two pseudo-subjects that were unknown to one another were simultaneously informed about the details of the experiment procedure. The subject and the pseudo-subjects did not converse with one another prior to the formal experiment, in which 232 trials were performed. The two pseudo-subjects performed the task on personal computers, whereas the true subject was positioned in the MR scanner. 

**Figure 1:**
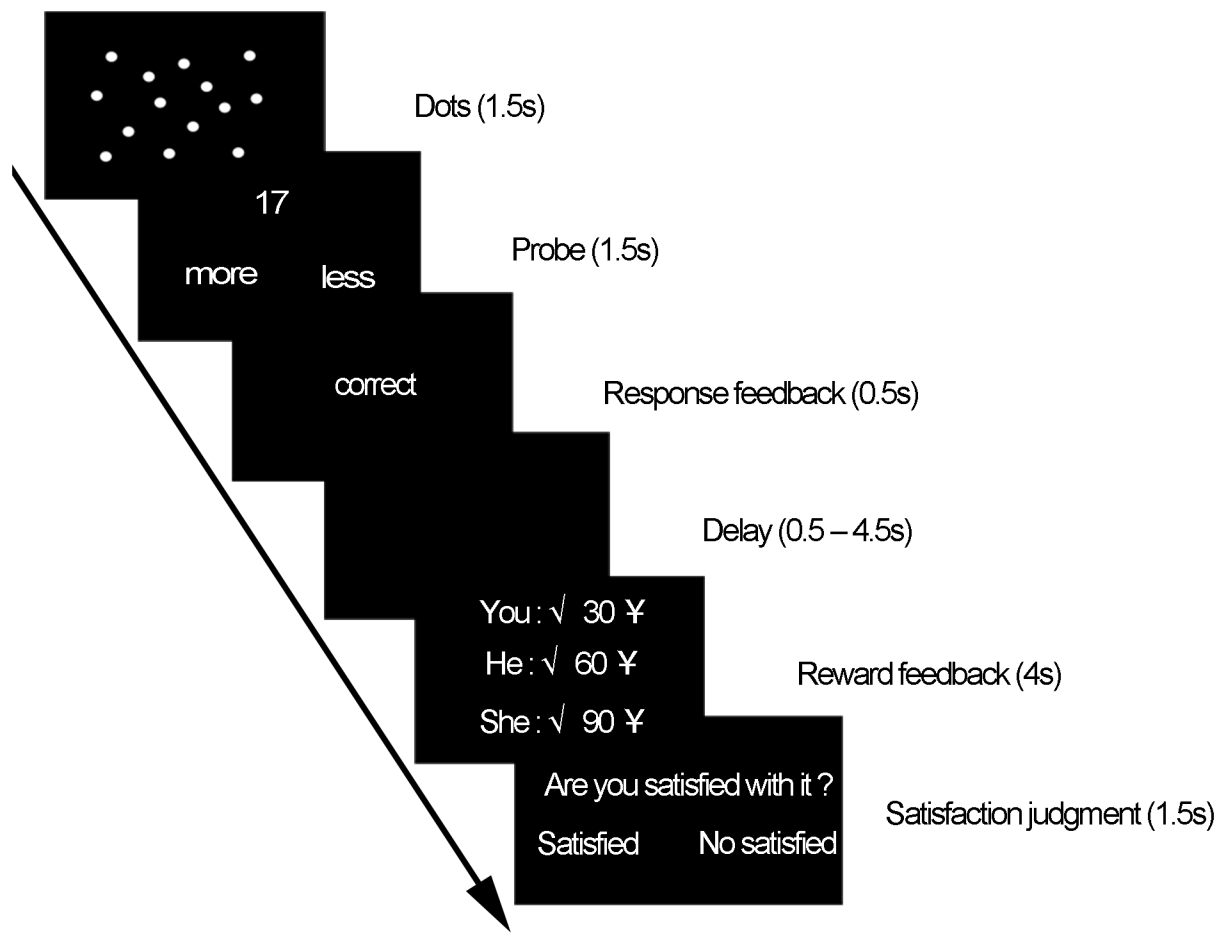
Single trial time-course (see Fig. 1 in the study of Fliessbach et al., 2007).

Prior to the experiment, all of the subjects were instructed to perform a simple number estimation task simultaneously. The performance and reward feedback of the two pseudo -subjects was predetermined. The time-course of a single trial is illustrated in Figure 1. The subjects were also instructed to view 10 to 40 white dots on a screen for 1.5 s. A numerical figure was then presented. The subjects were subsequently instructed to judge whether the number of white dots was lower or higher than the numerical figure by pressing key ‘3’ or ‘4’, respectively, on the fMRI keyboard by using the right hand. After a response feedback (0.5 s) and a short delay (0.5 s to 4.5 s), the subjects viewed a reward feedback screen containing information on their performance and their respective monetary rewards (4 s). Finally, the subjects were required to make a satisfaction judgment by pressing ‘3’ for ‘satisfied’ and ‘4’ for ‘not satisfied’. The next trial started after 0.5 s. The subjects were informed that the relative amounts of feedback were based on their relative response time (to enhance the subjects’ participation in this study by emphasizing the real outcomes and money that would be given according to their own performances, thereby reducing the subjects' feeling of being treated differently for the same performance). Thus, the subjects could potentially earn the maximum amount of monetary reward by responding as quickly and accurately as possible. 

Similar conditions were used for the practice program and the formal experiment to ensure that the number of trials under each condition was sufficient for analysis and mimic real-life situations. The response feedback was also predetermined. The reward conditions are illustrated in Table 1. Prior to the formal experiment, the subjects randomly specified an amount (120 Yuan, 180 Yuan, or 240 Yuan) according to the response in each trial. The conditions C1, C2, C3, and C4 were considered fair distribution, and C5, C6, C7, C8, and C9 were considered unfair distribution. To establish a more realistic experiment, we set a control condition (C3), in which the three subjects answered incorrectly or only one of the three subjects answered correctly. We excluded this condition from the statistical analysis because C3 did not contribute to the objectives of this study. 

After scanning, each subject was instructed to complete two questionnaires, including the big five personality questionnaire [24] and social adaptation scale [25].

### fMRI acquisition

A 3-T Trio scanner (Siemens) and an eight-channel phased array coil were used to acquire high-resolution T1-weighted structural images (1 mm×1 mm×1 mm) for anatomical localization and T2*-weighted echo planar images (32 slices, 3 mm×3 mm×3 mm voxels, TR = 2000 ms, TE = 30 ms, flip angel = 90°, FOV = 192 mm×192 mm), slice gap = 0.6 mm).

### fMRI data analysis

Data analysis was performed using SPM8 software from the Wellcome Department of Cognitive Neurology, London (SPM8, www.fil.ion.ucl.ac.uk/spm/) which is implemented on MatLab 7.10.0 R2010a (MathWorks, Natick, MA). All the analysis was started from the appearance of the reward feedback. Scans were started from slice time corrected, then realigned, normalized into standard Montreal Neurological Institute (MNI) space via 12-parameter affine transformation, finally, all data were smoothed with an 8mm full width at half maximum (FWHM) Gaussian kernel, and finally filtered (high-pass filter set at 128 s, low-pass filter achieved by convolution with the hemodynamic response function). After preprocessing, statistical analyses for each individual subject were based on the fixed-effects general linear models (GLM) and analyses on the level of the group were based on random-effects models [26]. The resulting images had cubic voxels of 3×3×3mm. The BOLD responses were modeled as events convolved with the canonical hemodynamic response function in SPM8. For each condition (C1, C2, C3, C4, C5, C6, C7, C8, and C9), all trials were averaged to estimate BOLD responses. 

In the group random effects (second-level) analysis, subject-specific linear contrasts of these parameter estimates were entered in a series of one-sample t-tests, each constituting a group-level statistical map. Our main contrasts of interest were BOLD signal in response to assess the main effect of conditions between C1 and C2. This contrast was used to identify the reward-sensitive regions of the brain. To correct for multiple comparisons within these analyses [27], we generated a cluster-level significance threshold using the AlphaSim program in the REST software (http://www.restfmri.net/forum/REST V1.7). This Monte Carlo procedure (1000 simulations, Gaussian filter width = 8 mm, cluster connection radius = 5 mm) estimated that whole-brain cluster-level correction for multiple comparisons at p < 0.05 was achieved for our data by combining a voxel-level threshold of p < 0.05 with a minimum cluster size of 389 contiguous voxels. We used this as the significance threshold for all of our analyses involving fMRI data.  

 Bold signal was then created for each subject according to the reward-sensitive regions of the brain by using the MarsBaR program (MRC Cognition and Brain Sciences Unit, Cambridge, United Kingdom, MarsBaR 1.86, http://www.sourceforge.net/projects/) to extract fMRI data from all voxels within the clusters for the conditions C5, C6, C7, C8, and C9. A one-way ANOVA (in SPSS; SPSS Inc., Chicago, Illinois) was performed on the extracted data to determine significant fMRI signal changes among the unfair conditions.

## Results

Data were expressed as the mean ± s.e. Group differences were assessed by two-way ANOVA followed by Newman-Keuls post-hoc test. P < 0.05 was considered as the significant level of difference.

### Behavioral Performance

**Figure 2:**
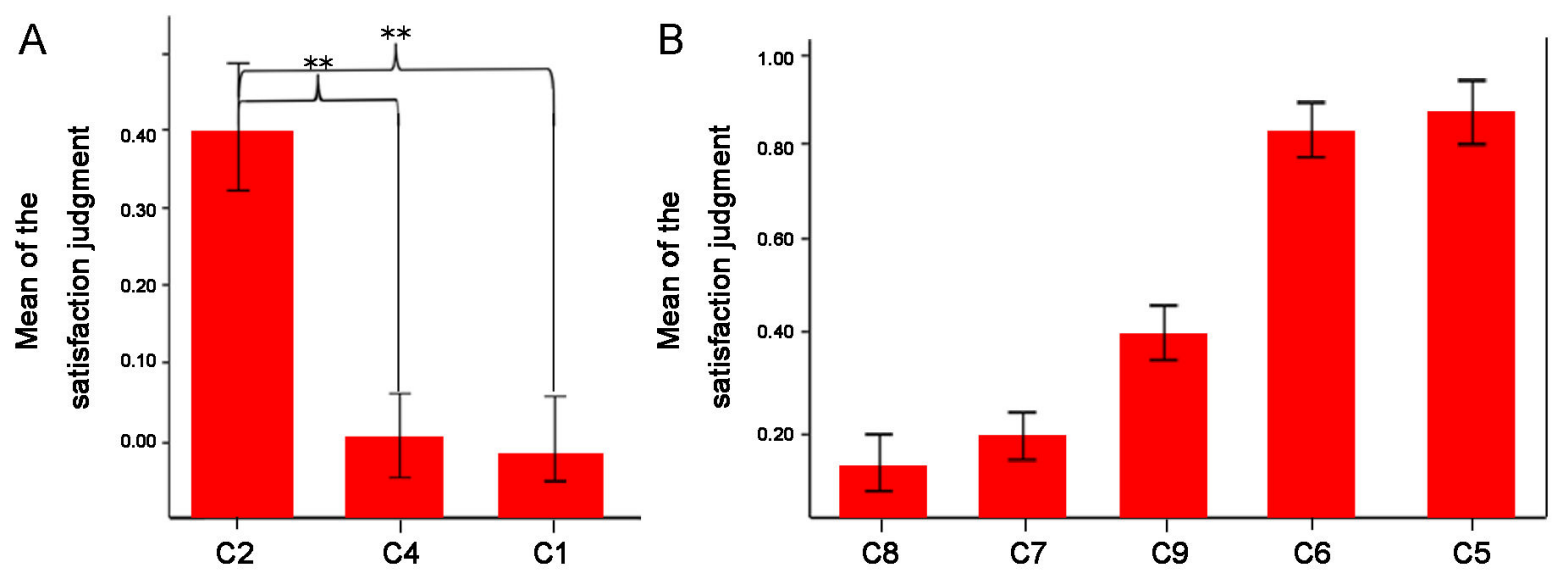
(A) Mean of the satisfaction judgment of the fair distribution conditions; (B) Mean of the satisfaction judgment of the unfair distribution conditions.

The mean of behavior rating on satisfaction judgment (the higher score means the more unsatisfied) for the fair conditions (C1, C2, and C4) were 0.01 ± 0.23, 0.506 ± 0.41, and 0.116 ± 0.24, respectively. Repeated measures ANOVA of the satisfaction judgment rates showed that the fair condition type exhibited a significant effect [F (2, 36) = 11.19, P < 0.001]. Pairwise comparisons showed that the subjects were more satisfied with C1 and C4 than with C2 (P<0.005; Figure 2). 

The mean of behavior rating on satisfaction judgment for the unfair conditions (C5, C6, C7, C8, and C9) were 0.882 ± 0.199, 0.819 ± 0.205, 0.185 ± 0.367, 0.131 ± 0.267, and 0.403 ± 0.399 (Figure 2). Repeated measures ANOVA of the satisfaction judgment rates showed that the unfair condition elicited a significant effect [F (4, 72) = 36.73, P < 0.001]. Post-hoc comparison results showed a significant difference between C5 and C6 as well as C8 and C9 (C6 is more satisfied than C5, C8 is more satisfied than C9), whereas no significant difference was observed between C7 and C8 as well as C7 and C9. 

### Functional Brain Activity

The condition in which the true subject was correct and received a reward and the two pseudo-subjects were not rewarded (C1) was compared with the condition in which the true subject was incorrect and all of the subjects were unrewarded (C2) to identify the reward-sensitive brain regions. This comparison significantly activated the Olfactory tubercle (ventral striatum: X, Y, Z =0, 17, -5), the Medial frontal gyrus (VMPFC: X, Y, Z = -6, 47, -11), the Superior frontal gyrus (DPFC: X, Y, Z = -15, 65, 10), the parahippocampal gyrus, and the left and right occipital cortexes, the left and right caudate, and the precentral gyrus (Table 2).

**Table 2:** Brain regions showing a significant BOLD-response for C1 vs. C2 (All regions are significant at *p* < .05 after whole brain cluster correction with voxel-level threshold of *p* < .05 and cluster size (*k*) of 389voxels.).

Brain region	MNI coordinates			Peak
	x	y	z	t-value
Caudate tail	36	-43	-2	6.62
Parahippocampal Gyrus	-21	-46	10	5.97
Middle occipital gyrus	-36	-91	10	5.42
Caudate	-3	20	-2	5.09
*Olfactory tubercle	0	17	-5	4.95
Caudate body	6	20	7	4.69
Cuneus	-6	-94	34	4.79
Superior occipital gyrus	-21	-88	31	3.39
*Medial frontal gyrus	-6	47	-11	4.76
Lingual gyrus	-24	-88	-17	4.06
*Superior frontal gyrus	-15	65	10	3.79
Precentral gyrus	18	-25	55	3.31

The regions marked ‘*’ are being used as regions of interest for the further analysis.

### Region of Interest (ROI) Analysis

**Figure 3:**
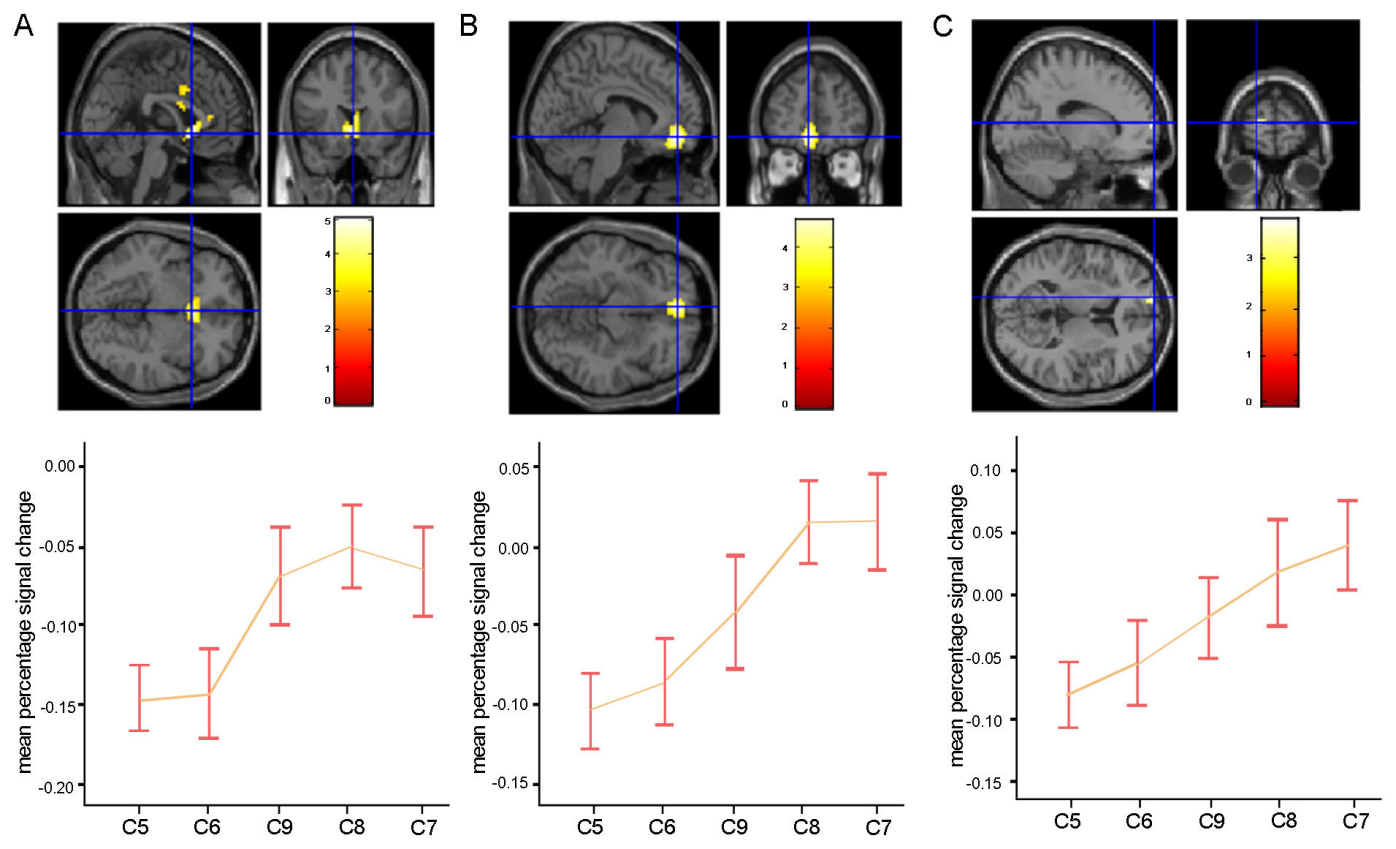
Effects observed in the brain in response to unfair distribution conditions: (A) Activation in the ventral striatum and the mean percentage signal change for the unfair distribution conditions in the ventral striatum; (B) Activation in the VMPFC and the mean percentage signal change for the unfair distribution conditions in the VMPFC; (C) Activation in the DPFC and the mean percentage signal change for the unfair distribution conditions in the DPFC.

ROI analysis was conducted in the ventral striatum, the VMPFC, and the DPFC to assess the function of these brain regions in unfair reward processing during social comparison. A significant effect of the unfair condition was observed in the ventral striatum, the VMPFC, and the DPFC (Figure 3).

One-way ANOVA of the responses to unfair conditions showed a significant effect in the ventral striatum [F (4, 72) = 6.01, P < 0.005]. The Newman-Keuls post-hoc comparison results showed that the ventral striatum was activated at a greater extent in C7, C8 and C9 than in C5 and C6. One-way ANOVA of the responses to unfair conditions showed a significant effect in the VMPFC [F (4, 72) = 5.16, P < 0.005]. Post-hoc comparison results showed no significant differences between C5 and C6, C5 and C9, C6 and C9, C7 and C8, C7 and C9, and C8 and C9. By contrast, a significant difference was observed between C5 and C7, C5 and C8, C6 and C7, and C6 and C8. One-way ANOVA of the response to unfair conditions also showed a significant effect in the DPFC [F (4, 72) = 4.51, P<0.01]. Post-hoc comparison results showed no significant differences between C5 and C6, C6 and C7, C6 and C8, and C6 and C9. By contrast, significant differences were found between C5 and C7, C5 and C8, and C5 and C9.

### Region-Region Relation

Different relationships are present between the ventral striatum, the VMPFC and the DPFC under unfair conditions. A significant correlation was also found between the percentage of signal changes in the ventral striatum and the VMPFC in C6 and C9 (C6, r = 0.535, P < 0.05; C9, r = 0.629, P < 0.005). A significant correlation was also observed between the percentage of signal changes in the VMPFC and the DPFC under these conditions (C6, r = 0.652, P < 0.005; C9, r = 0.456, P < 0.05). A significant correlation in the percentage signal changes in the VMPFC and the DPFC was observed only in C7 and C8 (C7, r = 0.556, P < 0.05; C8, r = 0.613, P < 0.01). By contrast, no significant correlations were observed in the three brain regions in C5. Under these conditions, r = -0.446 (P = 0.056) was obtained between the VMPFC and the DPFC. But we found there was no significant correlation in the ventral striatum and DPFC under all conditions.

### Brain-Behavior relation

The relationship between the ability to adapt to social pressure and the percentage of signal changes in the ventral striatum, the VMPFC, and the DPFC was determined. This assumption was investigated experimentally by using C9, in which the true subject experienced an unfair reward distribution. In C9, the true subject received less rewards than pseudo-subject but received more rewards than the other pseudo-subject. The scores of the social adaptation scale correlated significantly with the DPFC (r = -0.512, P < 0.05). No significant correlation was observed between the scores from the questionnaires and the percentage of signal changes in the brain regions.

## Discussion

The behavioral data observed under fair conditions showed that the true subject was more dissatisfied in C2 than in C1 and C4. However, the mean score of satisfaction judgment in C2 was approximately 0.5, indicating that dissatisfaction resulted from the interaction between the non-reward and the false response.

The behavioral data observed under unfair conditions showed that the true subject was more satisfied with in C7, C8, and C9 than in C5 and C6. The difference in the reward value among these five unfair conditions should be noted. For example, C5 is an unfair condition that the two pseudo-subjects were all the same amount and more than the true subject, so that there were two same negative value rewards of this condition. However, two different negative reward values were observed in C6, even though all the two pseudo-subjects were more than the true subject but there was a difference of the amount between the two pseudo-subjects. In C9, one positive value and one negative value were observed which means one pseudo-subject was more than the true subject and the other is less than the true one. In C7 and C8, the true subject received the highest reward. Therefore, the success of upward comparison showed that the differences in the reward values between C7 and C8 were irrelevant. No significant difference was detected in the satisfaction judgments between these individuals. 

Under the conditions in which the true subject received less rewards than one pseudo-subject and more rewards than the other pseudo-subjects, the setback resulting from upward comparison was limited by the satisfaction of the positive reward value. 

In C5 and C6, the true subject received the lowest reward. The absence of downward comparison in these conditions resulted in dissatisfaction. Therefore, the difference in the negative reward value was relevant and the true subject was more satisfied in C6 than in C5. However, the changes in the negative reward value did not result in the same level of satisfaction as in C9. 

The behavioral data presented in this study indicated that upward comparison is the primary response. However, associated negative emotions led to downward comparison to maintain self-evaluation in situations when this primary response failed. This response represents a stress coping mechanism similar to that reported in previous studies [10-12].

The results of fMRI and ROI analysis showed that the activities of the ventral striatum, the VMPFC and the DPFC were significantly different under the five unfair conditions. 

Higher activity in the ventral striatum was observed in C7, C8, and C9 than in C5 and C6. No significant differences were observed between C7, C8, and C9 or between C5 and C6. 

The percentage of signal change in the VMPFC in C7 and C8 was higher than that in C5 and C6. Furthermore, higher activation of the DPFC was observed in C8 than in C9, but C9 induced a higher activation of the same brain region than C5. Significant positive correlations between these brain regions were observed only in C6 and C9. C6 and C9 shared two common points: (1): the true subject was frustrated in the upward comparison and (2) the two reward values differed between the two conditions. 

Previous studies reported that the activity of the midbrain-ventral striatum dopaminergic projections is influenced by primary rewards such as sweet food and abstract forms of reward such as money or tokens [28-30]. Receiving an inferior reward was associated with a decrease in the percentage of signal change in the ventral striatum [21,31]. McClutre et al. [32] demonstrated that the activation of the ventral striatum is associated with the expectation of monetary reward. The activation of VMPFC is also observed from distributed fMRI patterns when expected values are decoded [33]. Büchel et al. [34] observed that VMPFC sends affective information about possible options based on reward values. By contrast, many studies have demonstrated that the DPFC is involved in rational processing [35,36] and utilitarianism (aggregate cost-benefit analysis) in social moral judgment [37-39]. This information further indicates that future behavioral acts should be induced based on appropriate reward valuation. 

In this study, the three subjects responded appropriately and the true subject anticipated receiving rewards that were not inferior to the other participants (pseudo-subjects). In C9, the negative reward value of upward comparison induced negative emotions, and this response was associated with the activity in the three brain regions. This activity resulted in the transfer of attention of the true subject from upward comparison to downward comparison. Thus, the reward value was converted from negative to positive to maintain self-value. This result also indicated that the DPFC is possibly involved in rational and utilitarian regulation of the reward circuitry. Furthermore, the VMPFC is possibly involved in the representation and conversion of such different reward values. The interaction between the DPFC and the VMPFC allowed the true subject to make a utilitarian choice in social comparison. The ventral striatum then adjusted the expectation of the reward. Therefore, these regions are responsible for the stress-coping mechanism reported in previous studies [11,12]. C6 differed from C9 in that blood oxygenation level-dependent (BOLD) signals in the ventral striatum and judgment were not satisfactory although activity was detected. This regulation may fail as a result of the absence of a true downward comparison target. However, the difference in the two negative reward values remains unclear in this study. In particular, regulation did not achieve values conversion and did not mitigate the feelings of setback.

The ability to adapt well in society requires effective self-regulation; C9 was similar to daily life experiences. A negative correlation was observed between the scores of social adaptation scale and DPFC activity. This result suggested that the DPFC of the subjects with a superior ability to adapt to social pressure does not require higher activity to modulate the reward circuitry and accomplish value conversion.

Behavioral data and the fMRI data also suggested that social context or social comparison is an important factor in the complex process of reward processing which requires the activation of multiple brain regions. The connection among the DPFC, the VMPFC, and the ventral striatum is crucial in the social comparison of reward processing, particularly in self-regulation of the setback resulting from upward comparison. Therefore, this study provided evidence of the interaction between the DPFC and the VMPFC. However, several studies have shown a dissociation between the DPFC and the VMPFC in cognitive and affective processing [38,40,41], although little information is available on the functional interaction between these brain regions.

The present study might be the first one using fMRI to investigate the neural mechanism in complex comparison including two confederates rather than one. Results showed that the ventral striatum and the prefrontal cortex might be related to reward processing under complex social comparison. PFC might played a role of self-regulation in the condition when there were two different reward values and the subject failed to exhibit upward comparison. This might be a possible supplement of the mechanism of stress-coping. However, there were still some shortcomings in our study. First, even though we want to make a more real situation of social comparison including two pseudo-subjects like the experiment of Fliessbach et al. (2007), there were two subjects really simultaneously and repeatedly performed a simple work task in two adjacent MRI scanners, while we just told subjects that they would perform a simple number estimation task with the other subject in the next laboratory simultaneously. The true subject did never converse with each other prior to the formal experiment. To some extent, this design would partly reduce the credibility of the experiment to the subjects. Second, due to the small sample size, so the current results only provided a model and tendency, rather than empirical data. In addition, the fMRI technology and this research had some inevitable limitations. Therefore, further studies should be done using both ERPs and fMRI to investigate spatiotemporal cortical activation patterns underlying the brain mechanism of context-dependent reward processing.
